# Role of Glutathione Peroxidase 4 in Glutamate-Induced Oxytosis in the Retina

**DOI:** 10.1371/journal.pone.0130467

**Published:** 2015-06-17

**Authors:** Osamu Sakai, Takatoshi Uchida, Murilo F. Roggia, Hirotaka Imai, Takashi Ueta, Shiro Amano

**Affiliations:** 1 Department of Ophthalmology, University of Tokyo of Medicine, Tokyo, Japan; 2 Senju Laboratory of Ocular Sciences, Senju Pharmaceutical Co., Ltd., Kobe, Japan; 3 School of Pharmaceutical Sciences, Kitasato University, Tokyo, Japan; 4 Inouye Eye Hospital, Tokyo, Japan; 5 Miyata Eye Hospital, Miyazaki, Japan; International University of Health and Welfare, JAPAN

## Abstract

**Purpose:**

The purpose of the present study was to investigate the role of glutathione peroxidase 4 (GPx4) in glutamate-induced oxytosis in the retina.

**Methods:**

For *in vitro* studies, an immortalized rat retinal precursor cell line R28 was used. Cells were transfected with siRNA specifically silencing GPx4 or with scrambled control siRNA. Lipid peroxidation was evaluated by 4-hydroxy-2-nonenal (4-HNE) immunostaining. Cytotoxicity and cell death were evaluated using an LDH activity assay and annexin V staining, respectively. Cells transfected with GPx4 siRNA or control siRNA were treated with glutamate (1 or 2 mM), and the cytotoxicity was evaluated using the LDH activity assay. For *in vivo* studies, retinal ganglion cell damage was induced by intravitreal injection of 25-mM N-methyl-D-aspartate (NMDA, 2 μL/eye) in GPx4^+/+^ and GPx4^+/−^ mice. The evaluation of lipid peroxidation (4-HNE immunostaining), apoptosis (TUNEL staining), and cell density in the ganglion cell layer (GCL) were performed at 12 h, 1 day, and 7 days after the NMDA injection.

**Results:**

GPx4 knockdown significantly increased LDH activity by 13.9-fold (P < 0.01) and increased peroxidized lipid levels by 3.2-fold in R28 cells (P < 0.01). In cells transfected with scrambled control siRNA, treatment with glutamate at 1 or 2 mM did not increase LDH activity; whereas, in cells transfected with GPx4 siRNA, glutamate treatment significantly increased LDH activity (1.52-fold, P < 0.01). GPx4^+/−^ mice exhibited higher levels of lipid peroxidation in retinas treated with NMDA than GPx4^+/+^ mice (1.26-fold, P < 0.05). GPx4^+/−^ mice had more TUNEL-positive cells induced by NMDA in GCL (1.45-fold, P < 0.05). In addition, the cell density in GCL of GPx4^+/−^ mice was 19% lower than that in GPx4^+/+^ mice after treatment with NMDA (P < 0.05).

**Conclusion:**

These results suggest that defective GPx4 expression is associated with enhanced cytotoxicity by glutamate-induced oxytosis in the retina.

## Introduction

Glutamate-induced neurotoxicity has been studied for its possible role in the pathogenesis of numerous neurological disorders, including Alzheimer’s disease, Parkinson’s disease, amyotrophic lateral sclerosis, and ischemic stroke [1]. Glutamate-induced toxicity may also be implicated in the ocular neurodegenerative changes in glaucoma [2–5] and diabetic retinopathy [6]. In fact, several studies have reported an increase in glutamate levels in the vitreous of patients with glaucoma [2] and proliferative diabetic retinopathy [7,8]. Because excess extracellular glutamate induces oxidative stress and cell death, glutamate-induced neurotoxicity is commonly called “oxytosis [1].” Treatments with antioxidants ameliorated the progression of the mouse model of glaucoma [9,10] and diabetic retinopathy [11] and suppressed cytotoxicity in retinal ganglion cells (RGCs) induced by N-methyl-D-aspartate (NMDA), the selective agonist for the glutamate receptor (NMDA receptor) [12,13]. Furthermore, treatment with an antioxidant suppressed the elevation of glutamate levels in the retinas of diabetic rats [14]. In addition, several studies have suggested the importance of endogenous antioxidative defense mechanisms, including a superoxide dismutase and thioredoxins in RGCs [15,16].

In glutamate-induced oxytosis, elevated levels of extracellular glutamate or increased susceptibility to extracellular glutamate can induce glutathione depletion and lipid peroxidation [1]. Among antioxidant enzymes, glutathione peroxidase 4 (GPx4) can directly reduce complex lipid hydroperoxides that are incorporated in biomembranes or lipoproteins [17]. We have elucidated the roles of GPx4 in photoreceptors [18], retinal pigment epithelium [19], and conjunctival cells [20]. Systemic abrogation of GPx4 leads to lethality on embryonic day 7 [21], and studies have identified drastic disease phenotypes of photoreceptors [18], cerebral neurons [22], vascular endothelium [23], and spermatocytes in conditional knockout mice [24].

In the present study, we evaluated the role of GPx4 in glutamate-induced oxytosis in the rat retinal precursor cell line R28 and the mouse retina.

## Methods

### Cell culture and transfection of siRNA

The rat retinal precursor cell line R28 was a kind gift from Dr. Yoshiaki Kiuchi (Hiroshima University, Department of Ophthalmology and Visual Sciences). R28 was established by immortalization of rat neuroretinal tissue at postnatal day 6 using the psi2 replication incompetent retroviral vector [25]. Unlike the RGC-5 cell line, R28 cells have been confirmed for validity and shown to express a variety of retinal cell-type markers, including RGC markers [26, 27]. The R28 cell line is considered suitable for neurotoxicity and neuroprotection studies [26]. Cells were cultured in Dulbecco’s modified Eagle’s medium (DMEM; Invitrogen, Carlsbad, CA) containing 10% FBS and 100 U of penicillin along with 100 μg/mL streptomycin under 5% CO_2_ at 37°C. Cells at 20–30% confluence were transfected with siRNA that specifically knockdown GPx4 and scrambled control siRNA (Ambion, Carlsbad, CA) using lipofectamine RNAiMAX (Invitrogen) according to the manufacturer's instructions.

### Real-time RT-PCR

Two days after transfection with GPx4 siRNA or scrambled control siRNA, total RNA was isolated using Isogen (Nippon Gene, Tokyo, Japan) according to the manufacturer’s instructions. For the *in vivo* studies, total RNA was isolated from microsurgically dissected mouse retinas in the same manner. Subsequently, RNA was reverse-transcribed into cDNA by the ReverTra Ace qPCR RT Master Mix with gDNA Remover (Toyobo, Osaka, Japan). Quantitative real-time PCR was carried out with the Thermal Cycler Dice Real-time System (Takara Bio Inc., Shiga, Japan) using Platinum SYBR Green qPCR SuperMix-UDG (Invitrogen). The values for each gene were normalized to the level of β-actin. The primer sequences used in the real-time RT-PCR were as follows: rat a-actin (Fwd, 5- CACCCGCGAGTACAACCTTC -3 and Rev, 5- CCCATACCCACCATCACACC -3), rat GPx4 (Fwd, 5- ATGCCCACCCACTGTGGAA -3 and Rev, 5- GGCACACACTTGTAGGGCTAGAGA -3), mouse GAPDH (Fwd, 5- CACATTGGGGGTAGGAACAC -3 and Rev, 5- AACTTTGGCATTGTGGAAGG -3), and mouse GPx4 (Fwd, 5- CGCGATGATTGGCGCT -3 and Rev, 5- CACACGAAACCCTGTACTTATCC -3).

### Immunoblotting

Two days after transfection with GPx4 siRNA or scrambled control siRNA, the proteins were extracted from cells and mouse retinas. SDS-PAGE of cellular proteins or retinal proteins was performed on Mini-PROTEAN TGX Any kD gel (Bio-Rad Laboratories, Hercules, CA) with Tris-glycine-SDS running buffer (Bio-Rad Laboratories). Immunoblot analysis was performed by electrotransfer of the proteins from the gels onto polyvinylidene difluoride (PVDF) membranes (Millipore, Billerica MA) at 100 V for 60 min at ice-cold temperature using Tris-glycine buffer. The membranes were probed with antibodies for β-actin (Santa Cruz Biotechnology, Dallas, TX) and GPx4 (Cayman, Ann Arbor, MI). Binding of secondary antibodies, conjugated to alkaline phosphatase or horseradish peroxidase, was observed using a chemiluminescent substrate (Pierce, Waltham, MA).

### Cytotoxicity assay

Two days after transfection with GPx4 siRNA or scrambled control siRNA, a cytotoxicity assay was performed using the lactate dehydrogenase (LDH) cytotoxicity detection kit (Takara Bio Inc.). LDH activity was measured in the extracellular medium and in the cell lysate, according to the manufacturer’s instructions; subsequently, extracellular LDH activity was calculated as a percentage of the total LDH activity. In the glutamate stimulation study, cells transfected with GPx4 siRNA or scrambled control siRNA were treated with 1-mM and 2-mM glutamate (Wako, Osaka, Japan). LDH activity was measured after 24 h.

### Determination of lipid peroxidation

Accumulated peroxidized lipids were assessed by immunohistochemical detection of 4-hydroxy-2-nonenal (4-HNE). Two days after transfection with GPx4 siRNA or scrambled control siRNA, cells were fixed with 4% paraformaldehyde for 15 min, washed three times with phosphate-buffered saline (PBS), and permeabilized with a 0.1% Triton X-100 solution containing 5% goat serum in PBS. Permeabilized cells were washed three times with PBS containing 5% goat serum and incubated with anti-4-HNE antibodies (JaICA, Shizuoka, Japan) overnight at 4°C. Cells were then washed three times with PBS. Alexa 488-conjugated anti-mouse IgG secondary antibodies (Invitrogen) were applied for 1 h at room temperature and washed three times with PBS. Fluorescent images were observed using a fluorescence microscope (Keyence, Osaka, Japan). The fluorescence intensities of the dots stained with 4-HNE were quantified using the Image J software (http://imagej.nih.gov/ij/; provided in the public domain by the National Institutes of Health, Bethesda, MD, USA).

### Detection of apoptosis

Two days after transfection with GPx4 siRNA or scrambled control siRNA, cells were stained by Alexa Fluor 488 annexin V (Invitrogen) for 15 min at room temperature and washed and rinsed with PBS. Fluorescent images were observed with a fluorescence microscope (Keyence). The percentages of annexin V-positive apoptotic cells relative to the total number of cells were calculated.

### Experimental Animals: GPx4^+/+^ and GPx4^+/−^ mice

We used GPx4^+/+^ and GPx4^+/−^ mice on the C57BL/6 background [19]. Specifically, all exons of GPx4 gene were replaced with a PGKneo cassette in the knockout allele [21]. Animals were maintained in ordinary animal cages under constant 12-h light/dark cycles. Food and water were available *ad libitum*. All animal experiments were performed in accordance with the Association for Research in Vision and Ophthalmology (ARVO) Statement for the Use of Animals in Ophthalmic and Vision Research and the NIH Guiding Principles in the Care and Use of Animals (DHEW Publication, NIH 80–23), and were approved by the Institutional Animal Research Committee of the University of Tokyo.

### Immunohistochemistry

Mice were sacrificed with an overdose of pentobarbital (100–150 mg/kg) injected intraperitonealy, and eyes were enucleated. Enucleated eyes of GPx4^+/+^ mice were fixed in 4% paraformaldehyde in PBS. The samples were paraffin-embedded and cut into 5-μm-thick sections. Slides were first incubated with blocking solution (2% normal goat serum) overnight and further incubated with anti-GPx4 antibodies at room temperature for 2 h and with Alexa 488-conjugated anti-mouse IgG secondary antibodies (Invitrogen) for 1 h. The sections were then coverslipped with mounting medium. Fluorescent images were observed using a fluorescence microscope (Keyence).

### NMDA-induced retinal toxicity

The intravitreal injection of NMDA was performed as described previously [4,5]. A total of 2 μl of 25-mM NMDA in PBS was injected into the vitreous body of GPx4^+/+^ and GPx4^+/−^ mice under anesthesia with intraperitoneal injection of a mixture of xylazine hydrochloride and ketamine hydrochloride.

The accumulation of peroxidized lipids in the retina was evaluated 12 h after intravitreal injection of NMDA. Mice were sacrificed with an overdose of pentobarbital (100–150 mg/kg) injected intraperitonealy, and eyes were enucleated. Then, eyes were fixed for 2 h in 4% paraformaldehyde solution in 0.1-M phosphate buffer (pH 7.4) and immersed for 1 h in PBS containing 20% sucrose. Further, the eyes were embedded in a supporting medium for frozen-tissue specimens (OCT compound; Tissue-Tek, Tokyo, Japan).

Retinal sections of 10-μm thickness were prepared using a cryostat at −25°C. Sections were immersed in PBS for 20 min at room temperature and incubated with anti-4-HNE antibodies (JaICA) overnight at 4°C. Sections were then washed three times with PBS. Alexa 488-conjugated anti-mouse IgG secondary antibodies (Invitrogen) were applied for 1 h at room temperature. The sections were washed three times with PBS and coverslipped with mounting medium. The intensity of immunofluorescence in the ganglion cell layer (GCL) and inner plexiform layer (IPL) was evaluated using the Image J software.

Retinal cell death was evaluated 24 h after intravitreal injection of NMDA. Mice were sacrificed with an overdose of pentobarbital (100–150 mg/kg) injected intraperitonealy, and eyes were enucleated. After fixation, the enucleated eyes were embedded in paraffin and incised through the optic disc of each eye at 3-μm thickness. TUNEL staining was performed according to the manufacturer’s protocol (In Situ Cell Death Detection Kit; Takara Bio Inc.) to analyze NMDA-induced cell death. Sections were treated with the TdT enzyme and stained with dUTP-fluorescein isothiocyanate. TUNEL-positive cells were observed using a fluorescence microscope (Keyence). The number of TUNEL-positive cells in GCL at a distance between 300 and 750 μm from the optic disc were counted.

Hematoxylin and eosin staining for morphological evaluation was performed 7 days after NMDA injection. Mice were sacrificed with an overdose of pentobarbital (100–150 mg/kg) injected intraperitonealy, eyes were enucleated, immersed for at least 24 h in 10% formalin, embedded in paraffin, and incised through the optic disc of each eye at 3-μm thickness. The sections were stained with hematoxylin and eosin, and photographed using a light-microscope. The number of cells in GCL was counted at a distance between 300 and 750 mm from the optic disc.

### Statistical Analysis

Data are expressed as mean ± SEM. Statistical analysis was performed with 2-tailed Student’s t-test. P < 0.05 was considered statistically significant.

## Results

### Effects of GPx4 knockdown in R28 cells

**Fig 1:**
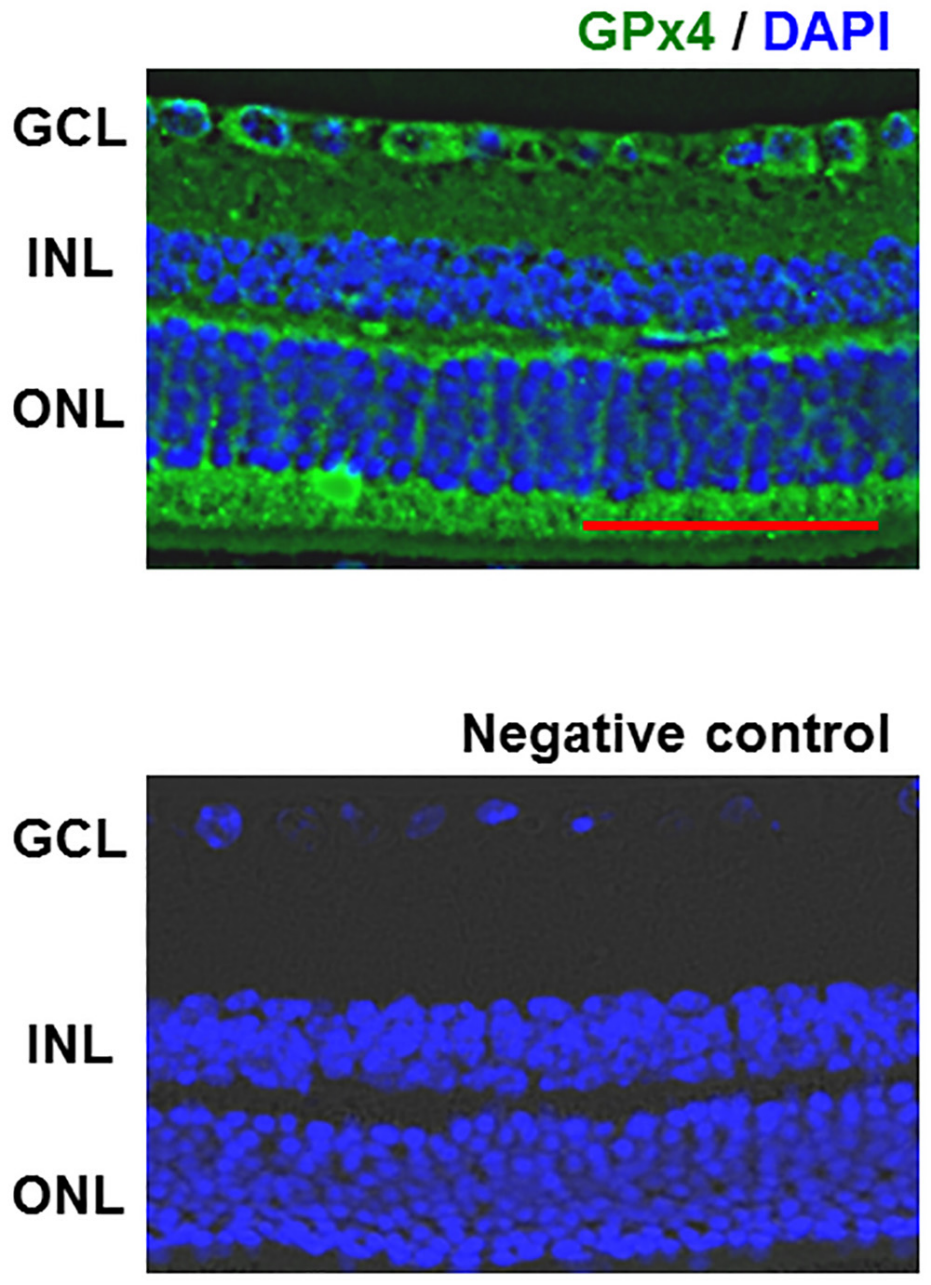
Glutathione peroxidase 4 (GPx4) expression in the mouse retina. GPx4 was ubiquitously expressed in the mouse retina except outer segments of photoreceptors. GCL = ganglion cell layer, INL = inner nuclear layer, ONL = outer nuclear layer, Scale bar, 50 μm.

**Fig 2:**
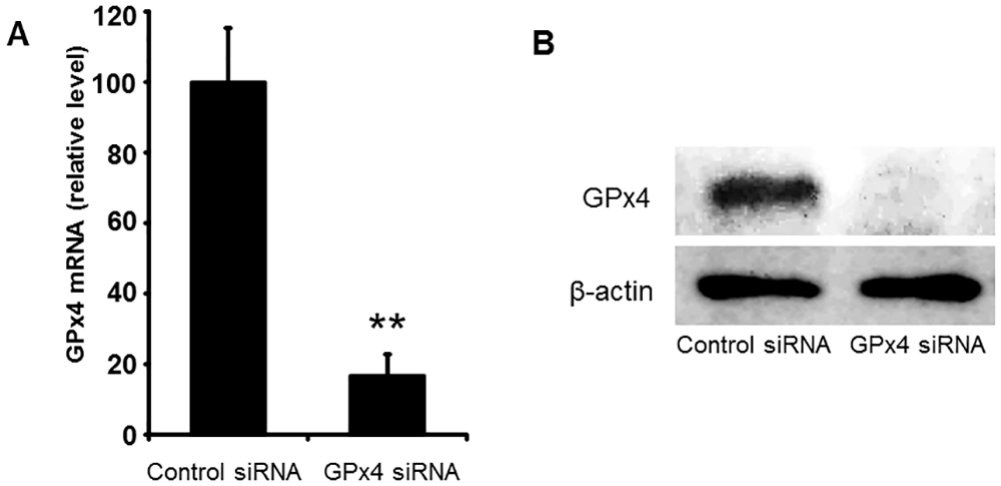
Knockdown efficacy of GPx4 in retinal precursor R28 cells. (A) The knockdown of GPx4 mRNA was confirmed by real-time RT-PCR. Data are mean ± SEM. (n = 3–4). **p < 0.01. (B) The knockdown of GPx4 protein was also confirmed by Western blot in triplicate.

First, we confirmed a ubiquitous expression of GPx4 in mouse retinas (Fig 1). The effects of GPx4 silencing in retinal cells were then evaluated using R28 cells. R28 cells were transfected with GPx4 siRNA to specifically knockdown GPx4. Two days after transfection, a favorable efficiency was confirmed at both mRNA (Fig 2A) and protein levels (Fig 2B) measured by real-time RT-PCR and Western blot, respectively.

**Fig 3:**
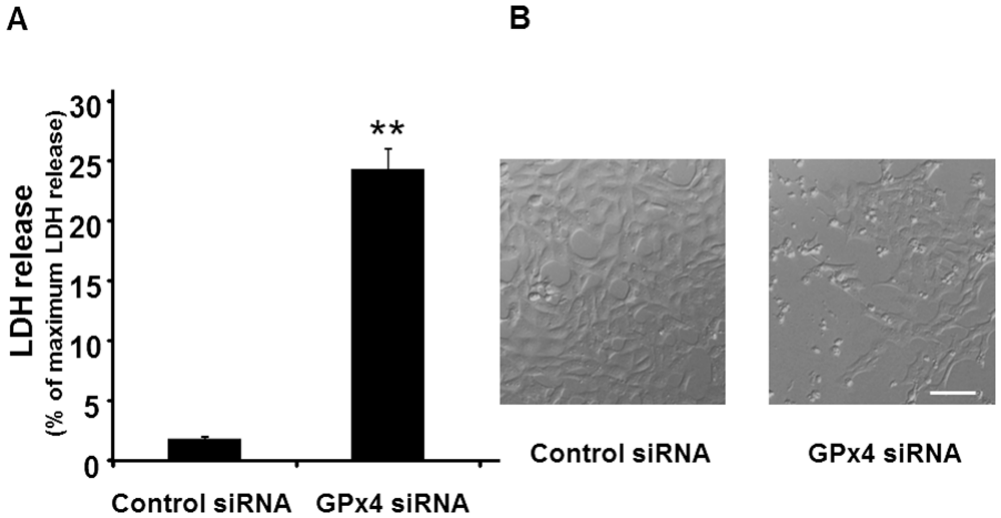
The effects of GPx4 knockdown on cytotoxicity in R28 cells. (A) LDH release from cells treated with control and GPx4 siRNA after 2 days of transfection. Data are means ± SEM (n = 4). **p < 0.01. (B) Phase contrast images of R28 cells after 2 days of transfection with scramble control or GPx4 siRNA. Scale bar, 50 μm.

Cytotoxicity was evaluated by measuring LDH activity. GPx4 knockdown significantly increased LDH activity (Fig 3A). Morphologically, cells treated with scrambled control siRNA appeared compact, uniform, and cobblestone-pavement shaped. On the other hand, cells treated with GPx4 siRNA exhibited signs of cell damage such as spheroid shapes (Fig 3B).

**Fig 4:**
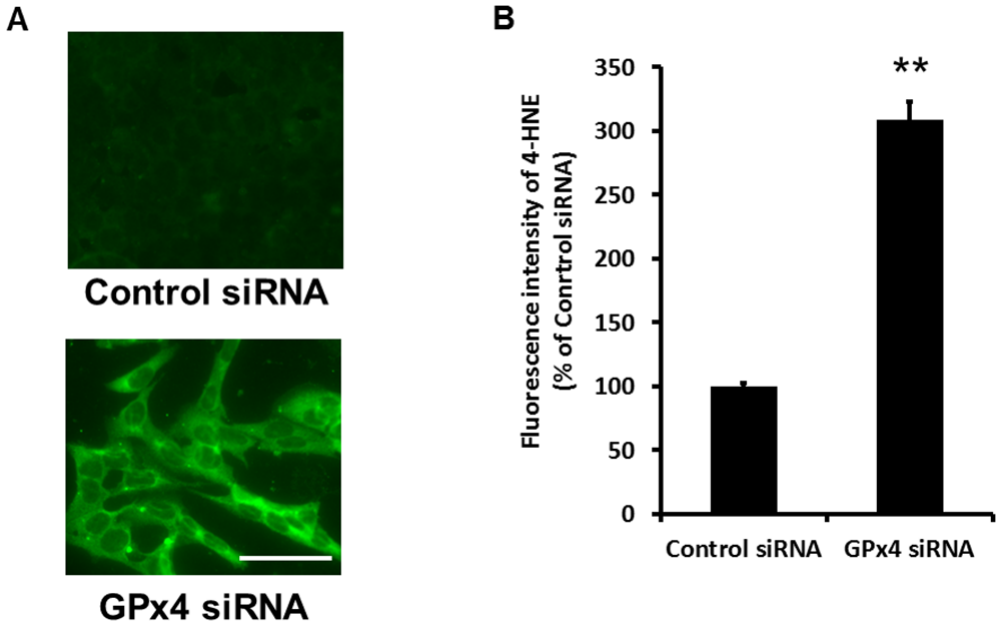
The level of peroxidized lipids in R28 cells. (A) 4-HNE detected by fluorescence microscopy using antibodies for 4-HNE. (B) Quantification of the fluorescence intensities for 4-HNE. Data are mean ± SEM (n = 4). **p < 0.01. Scale bar, 50 μm.

The accumulation of peroxidized lipids was evaluated by immunostaining of 4-HNE. 4-HNE immunostaining was three times higher in R28 cells transfected with GPx4 siRNA than in those transfected with scrambled control siRNA (Fig 4).

**Fig 5:**
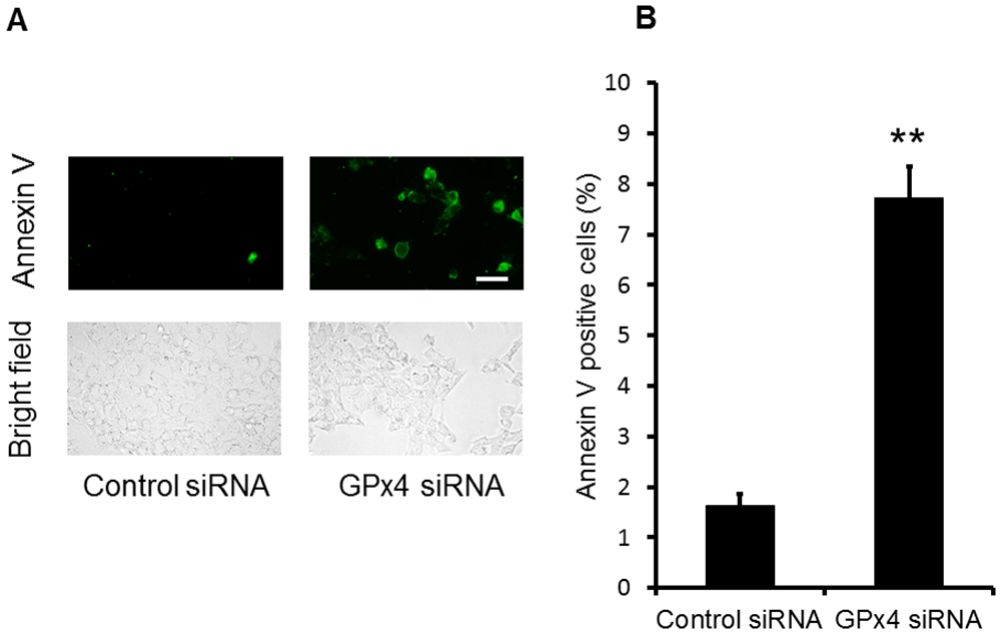
Apoptosis caused by knockdown of GPx4 in R28 cells. (A) Cells stained with annexin V by fluorescence microscopy. (B) The percentage of annexin V-positive cells relative to the total number of cells. Data are means ± SEM (n = 5). **p < 0.01. Scale bar, 50 μm.

Next, we evaluated apoptotic cell death using annexin V staining. As shown in Fig 5, the number of annexin V-positive cells significantly increased in R28 cells transfected with GPx4 siRNA compared with those transfected with scrambled control siRNA.

**Fig 6:**
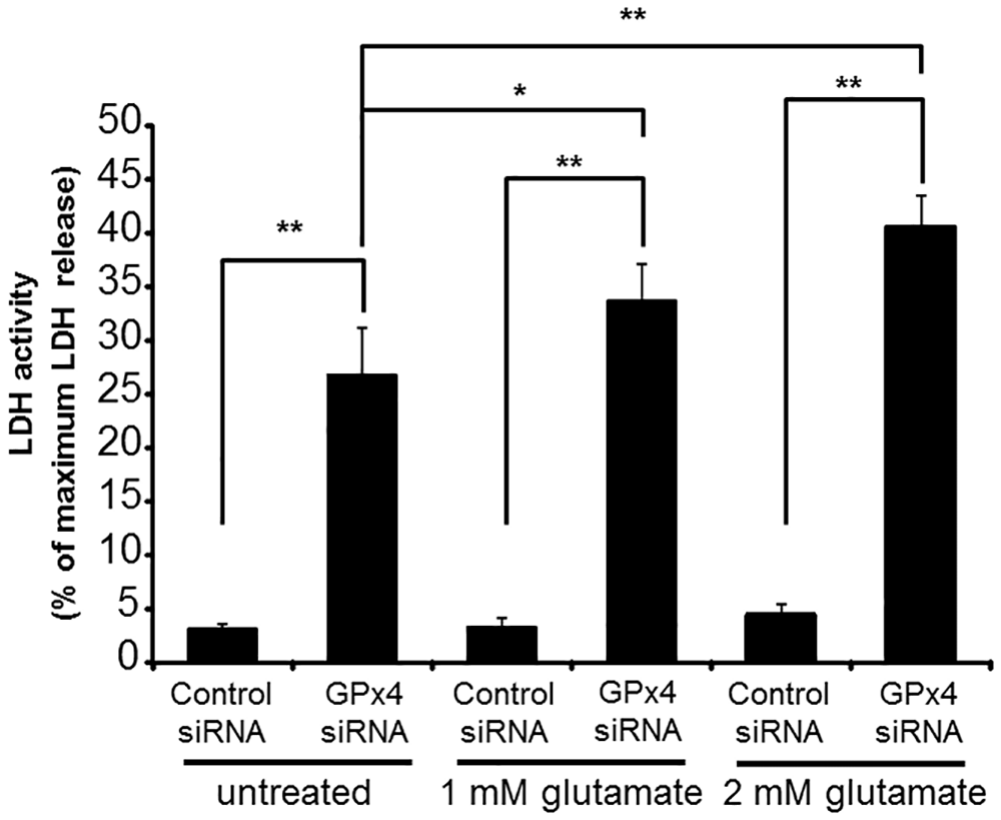
The effects of GPx4 knockdown on LDH release by glutamate cytotoxicity. LDH activity was evaluated after 24 h of glutamate treatment. Data are means ± SEM (n = 4). **p < 0.01.

We also investigated the effects of GPx4 knockdown on the cytotoxicity induced by glutamate (Fig 6). LDH activity of cells transfected with scrambled control siRNA was not influenced by glutamate up to 2 mM. However, GPx4 knockdown significantly enhanced the cytotoxicity induced by glutamate.

### NMDA-induced neurotoxicity in the retina of GPx4^+/+^ and GPx4^+/−^ mice

**Fig 7:**
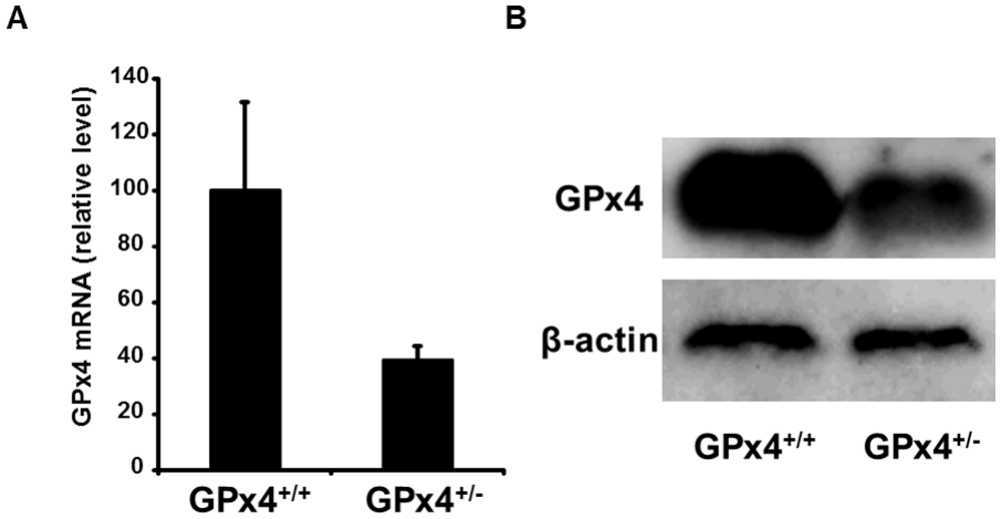
Gpx4 expression in the retina of GPx4^+/−^ and GPx4^+/+^ mice. (A) mRNA level was quantified by real-time RT-PCR, and normalized to GAPDH mRNA level. Data are means ± SEM (n = 5–6). (B) Protein level was determined by Western blot. Reproducibility was confirmed in triplicate.

First, we confirmed the decreased expression of GPx4 in both mRNA and protein levels in the retina of GPx4^+/−^ mice compared with those in the retina of GPx4^+/+^ mice (Fig 7A and 7B).

**Fig 8:**
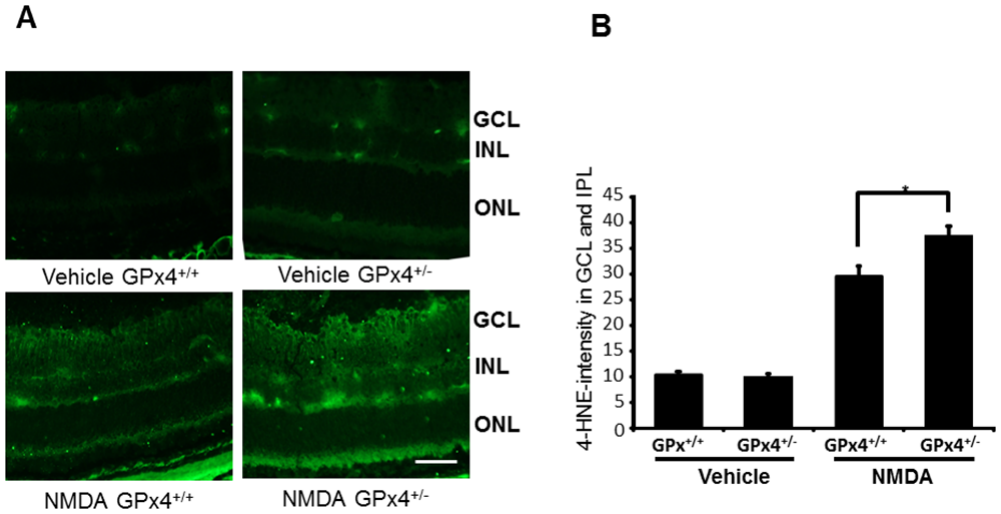
Lipid peroxidation in the inner retina (i.e., GCL and IPL) of GPx4^+/−^ and GPx4^+/+^ mice treated with vehicle or NMDA. (A) 4-HNE, as an indicator of lipid peroxidation, detected by immunohistochemistry using antibodies for 4-HNE. Scale bar, 30 μm. (B) Quantification of the fluorescent intensities of 4-HNE. Data are mean ± SEM (n = 9–10). *p < 0.05.

To evaluate the accumulation of peroxidized lipids, immunofluorescence for 4-HNE was measured in the inner retina (i.e., GCL and IPL). In vehicle-treated retinas of both GPx4^+/+^ and GPx4^+/−^ mice, 4-HNE immunoreactivity was rarely observed (Fig 8A and 8B). In contrast, although the retinas treated with NMDA exhibited an increase in 4-HNE immunoreactivity in both GPx4^+/+^ and GPx4^+/−^ mice, 4-HNE immunoreactivity in GCL and IPL was significantly higher in GPx4^+/−^ mice than in GPx4^+/+^ mice (Fig 8A and 8B).

**Fig 9:**
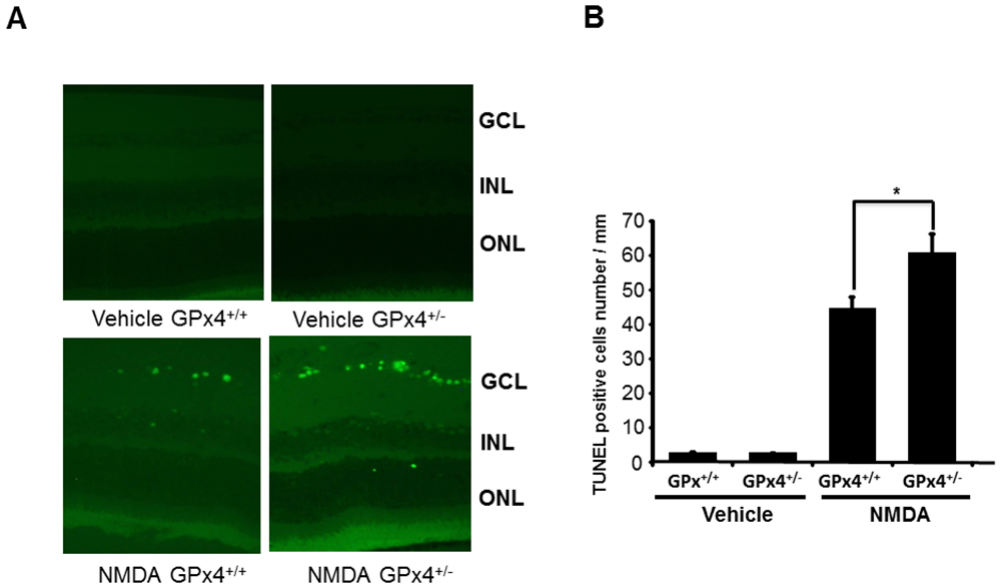
TUNEL staining in the retina of GPx4^+/−^ and GPx4^+/+^ mice treated with NMDA or vehicle. (A) TUNEL staining of the retina after NMDA or vehicle treatment. Scale bar, 30 μm. (B) Comparison of the number of TUNEL positive cells in the retina. Data are mean ± SEM (n = 9–10). **p < 0.05.

Next, we evaluated the extension of TUNEL-positive cell death in GCL 24 h after injections with NMDA or the vehicle. TUNEL-positive cells were hardly observed in the vehicle-treated retinas of both GPx4^+/+^ and GPx4^+/−^ mice (Fig 9). Intravitreal injection of NMDA induced TUNEL-positive cells in GCL of both GPx4^+/+^ and GPx4^+/−^ mice; however, the number of TUNEL-positive cells in GCL was significantly higher in GPx4^+/−^ mice than in GPx4^+/+^ mice (Fig 9A and 9B).

**Fig 10:**
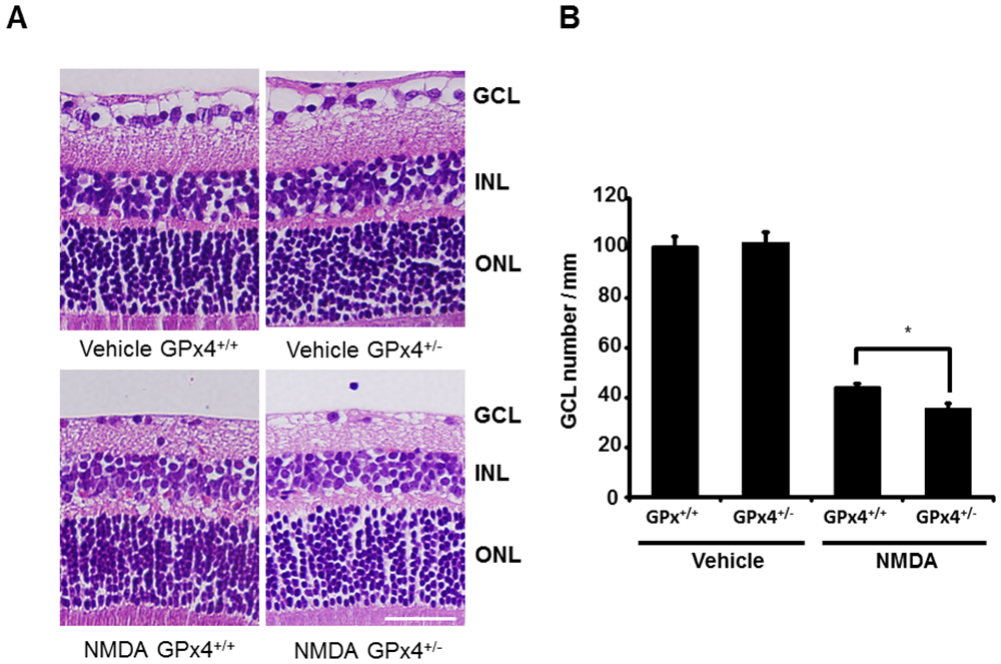
RGC loss in GPx4^+/−^ and GPx4^+/+^ mice after 7 days of NMDA or vehicle treatment. (A) Hematoxylin and eosin staining of retinal sections. Scale bar, 30 μm. (B) The number of cells in GCL were compared between of GPx4^+/−^ and GPx4^+/+^ mice. Data are mean ± SEM (n = 9–10). *p < 0.05.

Finally, as a result of the increased toxicity in NMDA-treated GPx4^+/−^ mice, we evaluated the number of remaining cells in GCL after 7 days of intravitreal injections with NMDA or the vehicle (Fig 10). There was no difference in retinal morphology and in the number of cells in GCL between vehicle-treated GPx4^+/+^ and GPx4^+/−^ mice. However, after NMDA treatment, the number of cells in GCL was significantly more decreased in GPx4^+/−^ mice than in GPx4^+/+^ mice.

## Discussion

The major findings of the present study are as follows: (1) GPx4 is an essential antioxidant enzyme for maintaining oxidative homeostasis in retinal cells. Decreased expression of GPx4 causes the accumulation of peroxidized lipids, cytotoxicity and apoptosis, as well as an increased susceptibility to glutamate toxicity *in vitro* and (2) GPx4 has an important role in protecting the retina from glutamate-induced oxytosis *in vivo*.

Extracellular glutamate inhibits cystine uptake into the cells via the cystine/glutamate antiporter, leading to the depletion of glutathione and accumulation of reactive oxygen species [1,28]. In glutamate oxytosis, glutathione depletion leads to lipid peroxidation through the activation of 12,15-lipoxyenase (12,15-LOX) [1]. LOX oxidizes polyunsaturated fatty acids (PUFAs) into lipid hydroperoxides, leading to the production of aldehydes, including 4-HNE. The accumulation of lethal levels of peroxidized lipids leads to mitochondrial damage and subsequent programmed cell death. Interestingly, 12,15-LOX has been shown to be involved in cell death in GPx4-deficient cells [29], which suggests a crosstalk between GPx4 and glutamate oxytosis. Consistently, in the present study we observed an increased susceptibility to glutamate cytotoxicity by silencing GPx4 in R28 cells, as well as increased peroxidized lipid accumulation and apoptosis in the retinas of GPx4^+/−^ mice compared with those of wild-type mice.

To date, several reports have suggested the essentiality and importance of antioxidant enzymes for the survival of RGCs. Yuki *et al*. have shown an increase in the NMDA-induced retinal neurotoxicity in SOD1-knockout mice [15]. Munemasa *et al*. transfected Trx1 and Trx2 genes by electroporation in the rat retina, and showed that RGC loss by elevated intraocular pressure was ameliorated in the retina transfected with Trx1 and Trx2 [16]. In addition to SOD and Trx, GPx is another major antioxidative defense mechanism, and to our knowledge, the present study is the first to reveal the role of GPx4 in the prevention of glutamate-induced oxytosis in the retina.

We used GPx4^+/−^ mice to address the effect of a decreased expression of GPx4 *in vivo* due to the embryonic lethality of GPx4^−/−^ mice and there is no established *Cre* mouse suitable for a conditional knockout in RGCs. Our results show that even 50% decrease in the expression of GPx4 causes severe damage in NMDA-treated retinas, suggesting the paramount importance of this antioxidant enzyme in the retina.

The implication of increased oxidative stress and decreased antioxidative capacity has also been studied in patients with glaucoma and diabetic retinopathy. In aqueous humor samples, decreased total antioxidative capacity and decreased concentration of vitamins have been observed in glaucoma patients [30,31]. Specifically, because vitamin E is important for the prevention of cell death caused by excessive lipid peroxidation and GPx4 deficiency [29], a decrease in aqueous humor [30] may be related to glutamate-induced oxytosis in the retina. In these reports, the activity of SOD and GPx was upregulated in aqueous humor of glaucoma patients [30,31], which was considered a compensatory mechanism. However, the measured GPx activity in aqueous humor might have reflected the activity of GPx3, the extracellular glutathione peroxidase, and not the activity of GPx4, the intracellular GPx. However, in the samples of peripheral blood cells, the activity of SOD and GPx was downregulated in glaucoma patients [32]. In diabetic patients with microvascular complications, the level of serum peroxidized lipids was upregulated while erythrocyte GPx and SOD activities were downregulated [33].

In conclusion, our data suggest that GPx4 is an essential antioxidant enzyme for protecting the neural retina from glutamate-induced oxytosis both *in vitro* and *in vivo*. Further studies are necessary to elucidate the role of GPx4 in the neurodegenerative changes in patients with glaucoma and diabetic retinopathy.
